# Supplementary material for: Differential contributions of synaptic and intrinsic inhibitory currents to speech segmentation via flexible phase-locking in neural oscillators
Source: PLoS Comput Biol. 2021 Apr 14;17(4):e1008783. doi: 10.1371/journal.pcbi.1008783 (PMC8104450; doi:10.1371/journal.pcbi.1008783)
Supplement: Attachment — Submitted filename: PLoS_Response_to_Reviewers_final.pdf [file pcbi.1008783.s007.pdf]

We heartily thank the reviewers for their incisive comments and suggestions. Guided by this feedback, we have undertaken a major revision, expanding the paper with several new analyses, and comprehensively rewriting the manuscript. Our intention with this revision has been to clarify the empirical status of our statements, and when possible to go further in testing and quantifying our claims.

The revised manuscript does more both to illuminate the mechanisms of phase-locking in our models, and to provide evidence of the utility of flexible phase-locking for speech segmentation. Towards the latter end, we have included a new analysis of the response of our models to auditory inputs derived from speech waveforms from the TIMIT corpus. This analysis shows that flexibility in phase-locking to synthetic rhythmic and quasi-rhythmic inputs translates to speech inputs, as well as showing a preference for phase-locking to mid-vocalic channels. Towards the former, we have included analyses that: test our hypothesis that the lower frequency limit of phase-locking depends on the delay until the first post-input spike; quantify the additive relationship between synaptic inhibition and the super-slow K current in model IS; and demonstrate the absence of this additive relationship, and the importance of STO phase, to post-input spiking in model MS.

Finally, guided by the reviewers' questions and our own exploration of the issues at stake, we have rewritten the Discussion section. Attempting to more comprehensively connect our work to vital issues in the neuroscience and linguistic of speech perception, we have drawn out the predictions and relevance of our results to auditory and speech processing and to cortical computation more broadly.

Reviewer #1: The manuscript describes a modeling work that explores the influence of different inhibitory currents on the phase locking properties of theta oscillations. The manuscript is well written, structured, and represents an interesting study, providing useful novel notions for future modeling in the domain of speech processing.

I do not have strong criticisms but a few points could perhaps be improved/specified.

Introduction:
What is the necessity of having a model phase-locked to rhythms slower than its intrinsic frequency? Since, as the authors suggest, there is no problem for the majority of models to lock to faster frequencies, a flexible oscillator could more easily be achieved by setting the intrinsic frequency at the lowest bound of the theta range.

We agree with the reviewer that this is a possibility, which we now address in the *Discussion* (line 396):

> *While an oscillator with an intrinsic frequency of 3 Hz might do an equally good job of phase-locking to strong inputs with frequencies between 3 and 9 Hz, this does not seem to be the strategy employed by the auditory cortex: the frequencies of (low-frequency) oscillations in primate auditory cortex are ~1.5 and ~7 Hz, not 3 Hz (Lakatos et al. 2005); existing experimental (Lakatos et al. 2005, Ghitza et al. 2007) and computational (Stanley et al. 2019) evidence suggests that cortical δ oscillators are unlikely to be driven at θ frequencies even by strong inputs; and MEG studies show that across individuals, speech comprehension is high when cortical frequencies are the same as, or higher than,*

speech envelope frequencies, and becomes poorer as this relationship reverses (Ahissar & Ahissar, 2005).

L25: I would not lump together beta/gamma (15-60 Hz) as it covers a range of diverse possible functions.

We agree that this frequency range covers a diversity of mechanisms and functions, and have amended the text to make note of this.

Results:
If, as the authors hypothesize, the model provides mechanism for flexible theta tracking, then the model should exhibit degradation of phase-locking at frequencies close to the upper bound of theta range. However, this is not clearly demonstrated in the results. Several candidate models still have high PLVs above 15 Hz, given a strong enough input strength.

We appreciate the reviewer raising this point. There are two clarifications relevant to this issue. First, while the PLV is high for frequencies above the theta band, it is not always one-to-one. I.e., for higher frequencies, the oscillator doesn't spike on each input cycle; some input cycles are "missed". We mention this in the *Discussion* section, but we have also added traces to Fig. 3 to show exactly where the boundaries of one-to-one phase-locking lie; this is now mentioned in the *Results* section, as well.

Secondly, as we now state in the *Discussion* (line 479):

> *Recent experiments shed light on the limits of adaptation to (uniform) speech compression, showing that while cortical speech-brain phase entrainment persists for syllabic rates as high as 13 Hz (a speed at which speech was unintelligible), β-rhythmic activity was abnormal in response to this unintelligible compressed speech [93]. This work suggests that the upper syllabic rate limit on speech intelligibility arises not from defective phase-locking, but from inadequate time for mnemonic or other upstream processes between syllables [93]. This agrees with our finding that the upper frequency boundary on phase-locking extends well above the upper syllabic rate boundary on speech intelligibility (~9 Hz), and is largely determined by input strength. Nonetheless, it is noteworthy that task-related auditory cortical entrainment operates most reliably over the 1-9 Hz (syllabic) ranges [73].*

Discussion:
L390: distinguish "additive" and "synergistic" more specifically?

We have substantially rewritten our discussion of these mechanisms, in both the *Results* section (*Dynamics of inhibitory currents*) and in the *Discussion* section. Beginning after line 268, we now write:

> *In models with a super-slow K current, this current, like synaptic inhibition, decayed to a nadir before each spike of the intrinsic rhythm. Unlike synaptic inhibition, IKSS*

activation built up dramatically during an input pulse (Fig. 7, green), and decayed slowly, increasing the latency of the first spike following the input pulse substantially (Fig. 2.3.1). This slow-building outward current interacted differently, however, with synaptic and intrinsic θ-timescale currents. In model IS, both Iinh and IKSS decayed monotonically following an input pulse, until the total level of hyperpolarization was low enough to permit another spike. We hypothesized that IKSS and Iinh interacted additively to produce hyperpolarization and a pause in RS cell spiking. In other words, the delay until the next spike is determined by the time it takes for a sum of the two currents' gating variables (weighted by their conductances and the driving force of potassium) to drop to a particular level. The fact that we expect this weighted sum of the gating variables to be nearly the same (having value, say, a∗) in the time t∗ before each spike suggests that the two gating variables are negatively linearly related:

$$gSOM{\rightarrow}RSs(t*)(V(t*) - EK) + gKSS\, q(t*)(V(t*) - EK) \simeq a*$$

~~this equation omitted here~~

Plotting the activation levels of these two currents in the timestep before each spike (excluding forced spikes and a handful of outliers) against each other confirmed this hypothesis (Fig. 8, model IS).

The interaction between Im and IKSS was more complex, as seen in model MS. The pre-spike activation levels of these two currents were not linearly related (Fig. 8, center). When IKSS built up, it dramatically suppressed the level of the m-current gating variable, biasing the competition between Im and INaP and reducing STO amplitude, and the IKSS activation had to decay to levels much lower than "baseline" before the oscillator would spike again. Indeed, spiking appeared to require m-current activation to return above "baseline", and also to be in the rising phase of its oscillatory dynamics. The dependence of spiking on the phase of the m-current activation could be seen by plotting the "phase plane" trajectories of the oscillator – plotting the m activation against its first difference immediately prior to each spike – revealing a branch of the oscillator's periodic trajectory along which pre-spike activation levels were clustered (Fig. 8, right). Plotting the second difference against the first revealed similar periodic dynamics (Fig. S2, top row).

These paragraphs refer primarily to the following figure:

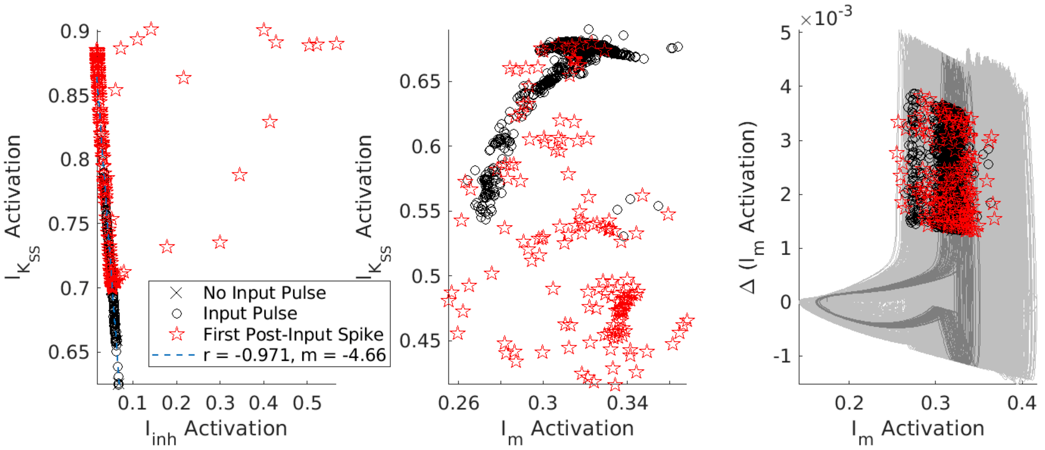

L403: the claim “neurons in deep cortical layers are likely to exhibit all three currents” need reference.

We thank the reviewer for catching this omission; it has been rectified by referencing Carracedo *et al.* 2013.

L455: ref missing?

And for catching this one, as well – a reference to an SfN abstract, which no longer appears in the manuscript.

L481 – 485: I think the authors mixed some numbers (average duration of a spoken syllable), or the reference (in the cited paper, they compress speech by x3 (40ms) chunks and inset silent gap from 0-120ms, the famous U-shape). The optimal performance there occurs when 40 ms speech chunk is followed by 80 ms silence chunk -> resulting in around 6Hz (120ms). Thus, they are right, if the average syllable duration is 333ms, then x3 compression would put the syllabic rate above 9Hz, and inserting silence according to U-shape (666ms?) would put it below 9Hz optimal rate. I am just having a problem, about where they take 333ms as average syllable duration. In the case of 200ms (5Hz), as reported in Greenberg (1999), and other studies, 3x factor would lead to 15Hz, out of theta range. In any case, I found this paragraph hard to follow, and rephrasing it would be desirable.

We appreciate the reviewer’s careful attention. We were in fact confusing results from two different references. To clarify, it would have been accurate to say:

The average spoken syllable lasts about 200 ms (Greenberg 1999), the period of a 5 Hz oscillation, and temporal compression by a factor of 3, increasing the syllabic rate to 15 Hz, results in a sharp drop in speech intelligibility (a word error rate around 50%). This decrement can be rescued by “repackaging” – inserting gaps of silence into the speech signal – with the highest comprehension occurring when segments of natural speech whose original (spoken) duration was 120 ms (corresponding to a frequency of ~8 Hz) are delivered at a rate of ~8 Hz (Greenberg 1999). Experiments using repackaging to estimate the human “auditory channel capacity” for speech – i.e., the maximum reliable information transfer rate – suggest it is ~9 Hz (Ghitza 2014).

However, in the revised manuscript, this passage no longer appears in the *Discussion*. We have, however, corrected and aligned the frequency ranges given for the theta rhythm throughout the paper (in so far as this is possible, given conflicting estimates in the literature).

L501: it would be helpful if the authors can suggest where these MS neurons are located in the auditory cortex.

We have done our best to explore the possibilities in the *Discussion* section, first starting on line 489:

More broadly, there is evidence that cortical θ oscillators in multiple brain regions, entrained to distinct features of auditory and speech inputs, may implement a variety of functions in speech processing. Different regions of human superior temporal gyrus (STG) respond differentially to speech acoustics: posterior STG responds to the onset of speech from silence; middle STG responds to acoustic onset edges (peaks in the rate of change of the speech amplitude envelope); and anterior STG responds to ongoing speech [98,99]. Similarly, bilaterally occuring δ/θ speech-brain entrainment may subserve hemispherically distinct but timescale-specific functions, with right-hemispheric phase entrainment [82] encoding acoustic, phonological, and prosodic information [31,82,88,100,101], and left-hemispheric amplitude entrainment [82] encoding higher-level speech structure [36,86,102,103] and top-down predictions [104–106]. Frequency flexibility may shed light on how these multiple θ oscillations are distinguished, collated, and combined. One tempting hypothesis is that the gradient from flexible to restricted phase-locking corresponds to a gradient from stimulus-entrained to endogenous brain rhythms, with oscillators closer to the sensory periphery exhibiting more flexibility and reverting to intrinsic rhythmicity in the absence of sensory input, enabling them to continue to couple with central oscillators that exhibit less phase-locking flexibility. It is suggestive that the conductance of the m-current, which is key to flexible phase-locking in our models, is altered by acetylcholine, a neuromodulator believed to affect, generally speaking, the balance of dominance between modes of internally and externally generated information processing [61,107–109].

Second starting on line 529:

Although from one perspective model MIS is the most physiologically realistic of our models, as neurons in deep cortical layers are likely to exhibit all three outward currents studied in this paper [48], the minimal impact of synaptic inhibition on these large pyramidal cells suggests that model MS is a functionally accurate representation of the majority (by number) of RS cells in layer 5 [61]. It thus represents the main source of θ rhythmicity in primary neocortex [61], and a major source of cortico-cortical afferents driving "downstream" processing [113,114]. Its properties may have strong implications for the biophysical mechanisms used by the brain to adaptively segment and process complex auditory stimuli evolving on multiple timescales, including speech.

Reviewer #2: The study by Benjamin Pittman-Polletta and colleagues addresses an interesting scientific question: how can neural oscillations be flexible enough to lock to quasi-rhythmic sensory signal such as the syllabic rate in speech? However, there are in my opinion several major shortcomings that severely limit the impact of the results (listed below). In the end, the study is stuck halfway between two possible outcomes: on one hand it does not give a theoretical account of how a neural oscillator can reliably lock down to an external input whose internal frequency fluctuates (although it features interesting phenomenological observations); on the other hand, there is no evidence that the novel model detects syllable boundaries better than existing models.

1 – From what I understood that the strategy to avoid missing one pulse is to

accumulate low time scales in neural dynamics in the oscillator. But then what is the point in using an oscillator in the first place, and not trivially a neuron that only reaches spiking threshold when a pulse is provided?

We now address this important question in the Discussion (starting on line 452):

> … *These considerations may explain why brain rhythms aid speech comprehension in noisy or otherwise challenging environments [83–85].*
>
> *This in turn suggests one answer to an important question raised by our results (and by one of our reviewers) – namely, if flexible entrainment to a (quasi-)periodic input depends on the length of the delay following input, why go to the trouble of using an oscillator at all, rather than a cell responding only to sufficiently strong inputs? The major difference between oscillators and non-oscillatory circuits driven by rhythmic inputs is what happens when the inputs cease (or are masked by noise): while a non-oscillatory circuit lapses into quiescence, an oscillator continues spiking at its endogenous frequency. Thus, oscillatory mechanisms can track the temporal structure of speech through interruptions and omissions in the speech signal [14]. This capability is crucial to the adjustment of speech processing to the speech rate, a phenomenon in which brain oscillations are strongly implicated: While (limited) speeding or slowing of entire utterances does not affect their intelligibility, altering context speech rate can change the perception of unaltered target words, even making them disappear [86–91]. In recent MEG experiments, brain oscillations entrained to the rhythm of contextual speech persisted for several cycles after a speech rate change [91], with this sustained rhythmic activity associated with altered perception of vowel duration and word identity following the rate change [91]. Multiple hypothetical mechanisms have been proposed to account for these effects: the syllabic rate (as encoded by the frequency of an entrained θ rhythm) may determine the sampling rate of phonemic fine structure (as effected by γ rhythmic circuits) [4,54]; predictive processing of speech may use segment duration relative to context speech speed as evidence to evaluate multiple candidate speech interpretations [44]; and oscillatory entrainment to the syllabic rate may time relevant (lexical, syntactic, and semantic) calculations, enabling the optimal balance of speed and accuracy in the passing of linguistic information up the processing hierarchy before the arrival of new input – so-called "chunk-and-pass" processing [92].*

2 – Syllable boundaries do not correspond to the high-energy vocalic portion of the syllable, but just the opposite: the low-energy portions corresponding to closure of the vocal tract. The vowel is the center (nucleus) of the syllable. Cutting a word such as "Badu" at high-energy portions would lead to 3 "syllables": "ba", "adu" and "u". Clearly not the most conventional definition of syllables…

We apologize for the confusion. We make the point in the revised manuscript that the syllables identified by our oscillator are the "theta-syllables" proposed by Ghitza (Ghitza 2013). These do not correspond to conventional linguistic syllables, but rather to the syllable-sized chunks that we and others propose the brain uses to segment and process speech on the syllabic timescale. These points have been clarified in the *Introduction* of the revised manuscript (starting at line 51):

> *… the lack of reliable acoustic markers of syllable boundaries, and the higher information density of the consonantal clusters that mark linguistic syllable boundaries relative to the high energy and long-duration vowels at their center, has led to the suggestion that reliable speech-brain entrainment may reverse the syllabic convention, relying on the high energy vocalic nuclei at the center of each syllable to mark segmental boundaries [4, 10, 14, 43, 60], enabling both robust determination of these boundaries and dependable sampling of the consonantal evidence that informs segment identity. These reversed "theta-syllables" are hypothesized to be the candidate cortical segments distinguished and passed downstream for further processing [14] by auditory cortical θ rhythms, but whether θ rhythms differentially entrain to different speech channels (associated with the acoustics of consonants and vowels) remains unexamined.*

We have also mentioned this in the *Discussion* of the revised manuscript, which now reads (starting at line 409):

> *Our models exhibit the highest level of phase-locking to the amplitude envelope of the mid-vocalic frequencies in speech, suggesting a pattern of entrainment to vocalic nuclei rather than consonantal clusters. These results provide empirical support for hypotheses suggesting that cortical θ oscillators align with speech segments bracketed by vocalic nuclei – so-called "theta syllables" – as opposed to conventional syllables, which defy attempts at a consistent acoustic characterization, but are (usually) bracketed by consonantal clusters [14]. These hypotheses are tied to theoretical frameworks (e.g. TEMPO [4,51]) proposing that pre-lexical chunking and sampling are mediated by a stimulus-entrainable cortical rhythmic hierarchy [41, 77, 81–83], consisting of γ and β rhythms whose amplitude is modulated by a concurrent θ rhythm in turn modulated by an ongoing δ rhythm [41,82]. This oscillatory hierarchy, whose timescales mirror the timescales of segments, syllables, and phrases in speech [17, 81, 82, 84–86], is believed to fit an adaptive (multi-scale) window structure to the acoustic signal, scaffolding multiscale processes of speech segmentation, sampling, and decoding [4,51]. … In this framework, "theta-syllables" have an information-theoretic advantage over conventional linguistic syllables: the vocalic nuclei of speech have relatively large amplitudes and durations, making them prominent in noise and reliably identifiable [17]; and windows whose edges align with vocalic nuclei center the diphones that contain the majority of the information for speech decoding, ensuring this information is sampled with high fidelity. These considerations may explain why brain rhythms aid speech comprehension in noisy or otherwise challenging environments [87–89].*

3 – It is not clear whether the proposed mechanisms allow better syllable detection than previous oscillator-based models of syllable detection (Hyafil et al, 2015; Räsänen et al., 2018). In particular, speech signal is far more complex than the input used here, with a spectrum likely dominated by 1/f component, so we would need to see how the proposed models behave in response to such signal. Second, it is not clear at all what level of phase-locking is required to accurately detect syllable boundaries, so it would really help to test actual syllable boundary detection, e.g. using the methodology developed in one of the above-mentioned studies.

We thank the reviewer for raising these deeply important issues. We have attempted, in the revised manuscript, to address the complexity of speech input by using sentences from the TIMIT corpus as inputs to our models. We show in the revised manuscript, using speech from the TIMIT corpus, that our oscillators show differential phase-locking to speech inputs filtered at different frequencies. This phase-locking is in agreement with our models' entrainment to rhythmic and quasi-rhythmic synthetic inputs.

The reviewer's questions about the relationship between syllable boundary detection and rhythmic entrainment are also vitally important. The "theta-syllable" hypothesis suggests that in fact, the brain avoids the inefficiency of attempting to determine *linguistic* syllable boundaries in speech, maximize its sensitivity to information-dense diphones by phase-locking to the amplitude envelope of auditory inputs at mid-vocalic frequencies. Our results support this hypothesis (see Fig. 5 and Section 2.2.3 of the revised manuscript).

How this phase-locking translates into syllabic segmentation is a subject of ongoing work, and unfortunately beyond the scope of the current manuscript, except for further elaboration in the *Discussion* section (starting at line 406):

> *How neural phase-locking to the speech envelope translates to the determination of segmental boundaries (if indeed this is a computational objective of speech processing), and to the temporal alignment of activity in downstream speech processing circuits with these boundaries, remains an open question. Out of our models, MS comes closest to spiking selectively at the peaks of the speech amplitude envelope, yet it does not perform perfectly. Its phase-locking to speech inputs is much poorer than its phase-locking to synthetic inputs. This is due in large part to the methodological challenge of accurately determining the phase of a signal as broadband and irregular as the amplitude envelope of speech (see Section 4.3.3), but it is also due to both "missed" and "extra" spikes (Fig. 5). Furthermore, phase-locking in model MS depends on tonic excitation, with lower levels of tonic excitation leading to more precise phase-locking but more missed cycles, and higher levels of tonic excitation leading to less precise phase-locking but a lower probability of any cycle being missed (Fig. S3). Whether the observed level of phase-locking is nonetheless functionally adequate for syllabic segmentation depends on a number of factors: on whether the inputs driving syllable-segmenting θ oscillators are pre-processed by other auditory cortical circuits; on whether syllable-segmenting θ oscillators tracking a given auditory channel or feature interact with other θ oscillators tracking the same or different channels or features; and on how syllable-segmenting θ oscillators drive other speech processing circuits. The tradeoff between precise and reliable phase-locking in model MS may be ameliorated in a network of such oscillators, connected synaptically or electrically, exhibiting heterogeneity in resting membrane potentials and/or input gains. The understanding of the dynamics of single (cell) oscillators presented here provides a necessary foundation for future investigations of network interactions.*

4 – The manuscript lacks clarity. A lot of things could/should be improved: it is very hard to follow the rationale for all the different mechanisms (the architecture seems completely arbitrary, until we get some intuition in Figures 5-6), as well as the specifics

of all 5 models; the Methods section is very difficult to follow as it is, placed before Results; Introduction section is too long; some elements are explained twice; some figures panels are not commented in main text (e.g. FI curves), some figure labels and panel labels are missing (e.g. Fig 2D), etc.

We apologize for this lack of clarity, and appreciate the reviewer's drawing our attention to it. We have attempted to remediate this issue with a wholesale rewriting of the manuscript. Among other changes, we have:

- placed the Methods section at the end of the manuscript;
- attempted to better motivate our experimental results, including and especially the architectures of the models, which are now also briefly mentioned in the *Introduction*;
- attempted to further clarify our discussion of the dynamics of inhibitory currents;
- removed extraneous summaries of the literature from both the *Introduction* and *Discussion* sections;
- highlighted the crucial empirical issues addressed by the paper in the *Introduction*;
- made efforts to remove redundancies in the text that do not add to clarity; and
- made efforts to expand the figure captions.

While we were unable to shorten the *Introduction*, we believe it now more clearly and directly states the aims, novelty, and significance, of the manuscript.

MINOR POINTS
- How do SOM neurons respond?

The conductance $g_{RS\rightarrow SOM}$ was tuned to preserve a one-to-one spiking ratio between RS and SOM cells during spontaneous (intrinsic) rhythmicity. As a result, aside from a larger percentage of noise-induced spikes, SOM cell responses are qualitatively similar to RS cell responses. This is now mentioned in the *Methods* (line 576).

- Is the architecture of SOM neurons taken from any existing reference?

It is taken from Lee *et al.* (2013), and we have included this information in the revised manuscript.

- What is chi(t) line 119?

We use chi_S(t) as notation for the indicator function that takes value 1 on set S and value 0 otherwise. In the previous version of the manuscript, we defined this later, on line 149; we have now moved this definition earlier to avoid confusion.

- A plot/inset of periodic pulses and quasi-period pulses would help.

In Figure 3, we have changed the location of the plot of periodic pulse input to make it easier to see, and changed the appearance of the plots of model voltage to make the response of each model easier to see. We have also added similar plots to Figure 4, and we show rastergrams of the model responses to a sample sentence from the TIMIT corpus in Figure 5.

- Why are baseline frequencies not lower for models with more inhibitory currents?

We have chosen the tonic input current so that all models exhibit the same baseline frequency. This allows us to naturally compare the range of frequencies at which different models exhibit phase-locking. This point has been clarified in the *Results* (starting on line 132):

> *For all simulations, we have chosen and fixed I_{app} so that all models exhibit intrinsic rhythmicity at the same frequency, 7 Hz (Fig. 1, inset FI curves, red circles), allowing us to compare the range of input frequencies over which different models exhibit phase-locking.*

- The Hilbert transform is a more principled method for extracting the phase of the input signals than the one used here.

Unfortunately, the Hilbert transform only gives reliable results for signals that are suitably "oscillation-like". Our frequency-modulated simulated speech inputs don't satisfy this property (nor do our speech inputs). These points have now been made in the *Methods* (line 605):

> *Since I_{VP} was composed of pulses and interpulse periods of varying duration, it was not "oscillation-like" enough to employ standard wavelet and Hilbert transforms to obtain accurate estimates of its instantaneous phase. Instead, the following procedure was used to obtain the instantaneous phase of I_{VP}. …*

(Line 629):

> *Like varied pulse inputs, speech inputs were not "oscillation-like" enough to estimate their instantaneous phase using standard wavelet and Hilbert transforms. Thus, we used the following procedure to extract the instantaneous phase of I_{speech}. …*

- Figure 1: why are there 2 FI plots for each curve?

For each model, the smaller FI plot is an inset, showing a "zoomed-in" version of the same FI plot, for a smaller range of input currents. We have modified the figure to make this clearer, using boxes superimposed on the larger FI plots (showing the extent of the inset plots) and lines between the large and small plots to indicate this relationship.

- Why use 'outward current' and not 'inhibitory current' consistently?

We use "outward current" occasionally in order to avoid the confusion that may result from the association of the word "inhibitory" with synaptic inhibition.

- The last sentence of the abstract mentions something about the neural oscillator allowing "reliable time-keeping", but I found no reference to this function in the manuscript.

The phrase “reliable time-keeping” was inappropriate to our meaning, and we have changed it to “reliable internal clocking”. The point we meant to allude to is addressed in the penultimate paragraph of the *Discussion*. We have changed the language in this paragraph to better tie in with the text of the abstract. It now reads (line 516):

> *Our results suggest that mechanisms like that of hippocampal θ, far too inflexible to perform the segmentation tasks necessary for speech comprehension, are instead optimized for a different functional role. One possibility is that imposing a more rigid temporal structure on population activity may help to sort “signal” from “noise” - i.e., imposing a strict frequency and phase criterion that inputs must meet to be processed, functioning as a type of internal clock. Another possibility is that more rigidly patterned oscillations result from a tight relationship to motor sampling routines which operate over an inherently more constrained frequency range, as, for example, whisking, sniffing, and running are related to hippocampal θ.*
