# Supplementary material for: Differential contributions of synaptic and intrinsic inhibitory currents to speech segmentation via flexible phase-locking in neural oscillators
Source: PLoS Comput Biol. 2021 Apr 14;17(4):e1008783. doi: 10.1371/journal.pcbi.1008783 (PMC8104450; doi:10.1371/journal.pcbi.1008783)
Supplement: Attachment — Submitted filename: Second_response_to_reviewers.pdf [file pcbi.1008783.s008.pdf]

We thank both reviewers for their keen eyes and kind words. In addition to making the suggested corrections, we took the second reviewer's comments about speech segmentation to heart. Thus, we have expanded our results on speech stimuli, adding new simulations that explore the functional consequences of our models' phase-locking behaviors for speech segmentation. In these new simulations, we use a simple mechanism to derive segmental boundaries, summing population spiking activity across time (and neurons), and applying a threshold to determine putative syllable boundaries. We also explore the phoneme distribution of these model-derived boundaries, and find that they lie predominately within vocalic nuclei. We use a well-established timing-based metric for computing the distance between spike trains, to compare these model-derived syllable boundaries to the midpoints of the syllables determined from the phonetic transcriptions of the TIMIT corpus. While not a direct comparison to previous methods of syllable segmentation, our results clearly show that the flexible phase-locking afforded by intrinsic outward currents enables the recovery of more accurate syllabic-timescale segmental boundaries, than the synaptic inhibition-based oscillators modeled by others in earlier work on speech segmentation, through consistent identification of vocalic nuclei. These new results have enlarged the story, and we have rewritten the abstract, introduction, and discussion to accommodate it. We hope the reviewers will find the new version improved (again), thanks in no small part to their contributions.

Below, we address the reviewers' other comments point-by-point.

Reviewer #1: The previous comments have been properly addressed, therefore I only have minor comments here.
L142: typo, "rage" range.

This has been corrected.

L201: "phase locking of model MS… by a lack of spiking when there is no speech input". This also seems to be the case for other types. How was this conclusion drawn? It would be helpful to have a quantitative metric, e.g. correlation between the speech envelope and instantaneous firing rate of the neuron.

As suggested, we examined the correlation between the speech envelope and the instantaneous firing rate. However, we found no differences among our models. Thus, we have removed this statement. While we still believe it may be true, we have not yet encountered the right metric or method to assess its veracity.

Fig. 6: need color bar for the phase locking values on the right-side plot.

Thanks; this has been added.

L456-457: "may time relevant calculations…" please correct.

We removed the parenthetical clause "(lexical, syntactic, or semantic)" from the sentence in question, which we believe was the reviewer's suggestion.

Reviewer #2: The revised manuscript constitutes in my opinion a major improvement in comparison to the original submission. It has gained a lot in clarity, concepts are much better articulated, and my major concerns have been addressed – or at least clarified. I am still quite uncomfortable with using the term "speech segmentation" in the title though, since really all the work is about measuring phase-locking and authors have not checked whether speech signal would be segmented in any meaningful manner. I also strongly suggest to openly report this in the Discussion as a limitation of the study: flexibility is very nice but it remains to be shown that such a model outperforms previous models of speech segmentation.

As mentioned above, we have included major additions in the revised manuscript to address this important issue.

Here is a list of minor comments below:

- Label for Y-axis is missing on figure 2B bottom

We have added a label. (For clarity, the left and right y-axes give the units for the m-current and the persistent Na current, respectively, as indicated by the legend.)

- Legend figure 2D: calibration means the scale of the horizontal and vertical bar?

Yes; the legend has been changed accordingly.

- L256: "each spike suggests that the two gating variables are negatively linearly related" -> specify: "at spike times"

Thank you for the suggestion; this has been added.

- Figure 8 is messy, legend says we should see no input pulse but I cannot see any 'x', the regression line is also hard to see, consider using smaller symbols or points. Missing parenthesis in capture.

We have made the symbols smaller for spikes from simulations with input pulses, and increased the line thickness of the regression line, which we believe helps with legibility.

- Sentence L301-307 is very long and difficult to understand, consider simplifying.

We have rewritten this sentence. It now reads:

> The fixed delay time of synaptic inhibition seemed to stabilize the frequency range of phase-locking, while the voltage-dependent and "elastic" dynamics of the m-current seemed to do the opposite. Specifically, the four models containing I_{inh} exhibited an intermediate frequency range of phase-locking, while both the narrowest and the broadest frequency ranges of phase-locking occurred in the four model θ oscillators containing I_m; and the very narrowest and broadest ranges occurred in the two models containing I_m and lacking I_{inh} (Fig. 3).

- Formulation of sentence L423-427 is awkward.

The sentence in question no longer appears in the current manuscript. The passage in which it appeared now reads (L597-607):

> Our results support the hypothesis that cortical θ oscillators align with speech segments bracketed by vocalic nuclei – so-called "theta syllables" – as opposed to conventional syllables, which defy attempts at a consistent acoustic characterization, but are (usually) bracketed by consonantal clusters [16]. These "theta-syllables" are suggested to have information-theoretic advantages over conventional linguistic syllables: the vocalic nuclei of speech have relatively large amplitudes and durations, making them prominent in noise and reliably identifiable [19]; and windows whose edges align with vocalic nuclei center the diphones that contain the majority of the information for speech decoding, ensuring this information is sampled with high fidelity. These claims, if they prove to have functional relevance, may illuminate how speech-brain entrainment aids speech comprehension in noisy or otherwise challenging environments [97–99].

- Chi(t) is still not explained when it is introduced L557.

Thanks for catching this omission; it has been corrected.

- Please explain the rationale for the change in applied current after 500 ms L557.

The applied current ramps up from zero during the first 500 ms to minimize the transients that result from a step current; this information has been added to the manuscript.

- Is the negative sign correct for Iext in equation L561?

Yes; the synaptic current I_{exc} is treated the same as other currents generated by membrane channels.

- L564: "conductance values for all six models that will be introduced in Results:" -> correct future tense

This has been corrected.
